# Entry inhibition of human retroviruses in rat cells

**DOI:** 10.1186/1742-4690-8-S1-A20

**Published:** 2011-06-06

**Authors:** Masahiko Shinagawa, Kaya Miyazaki, Xianfeng Zhang, Jing Chen, Takashi Ohashi, Hisatoshi Shida

**Affiliations:** 1 Institute for Genetic Medicine, Hokkaido University, Sapporo, 060-0815, Japan

## 

Human T cell leukemia virus type 1 (HTLV-1) and human immunodeficiency virus-1 (HIV-1) are representative human retroviruses. To develop prophylactic vaccines and more effective medicines, small animal models for the virus infection are useful if they can be infected with the viruses. We constructed a transgenic (Tg) rat expressing human CRM1 (hCRM1), a cellular cofactor of Rex, and showed that T cells derived from Tg rats allowed production of HTLV-1 as efficiently as human T cells. However, viral load in Tg rats was not elevated so much. Our trials of various infection methods including oral administration to neonates failed to improve viral load, suggesting another barrier. During exploration we found lower rate of infection to rat dendritic cells (DC) than human DC, and administration of cyclosporine A (CSA) and As2O3 restored the infection rates. These results suggest the presence of inhibitor(s) that act during the entry process in rat DC.

We also constructed Tg rats expressing human CD4, CCR5, CRM1, and CyclinT1, a cofactor of Tat to develop a model for HIV-1 infection. T cells and macrophages of the Tg rats supported production of infectious virus at levels approximately 10- 40% of that detected in human cells. HIV-1 was efficiently infected to macrophages but not to CD4+ T cells. CSA and As2O3 restored the infection rates, suggesting the presence of inhibitor(s) that act during the entry process in rat CD4+ T cells.

